# Supplementary material for: Does mental fatigue affect skilled performance in athletes? A systematic review
Source: PLoS One. 2021 Oct 14;16(10):e0258307. doi: 10.1371/journal.pone.0258307 (PMC8516214; doi:10.1371/journal.pone.0258307)
Supplement: S1 Table — (DOC) [file pone.0258307.s001.doc]

**S1 Table - Detailed search strategy**

|  | (P) Athletic population (I) fatigue (O) sport skills | Results |
| --- | --- | --- |
| Pubmed  (1946 – Jan 2021) | (("mental fatigue" OR "mental exertion" OR "cognitive fatigue" OR "cognitive exertion" OR "mental exhaustion" OR "mental tiredness") AND ("athletic performance" OR "technical skill*" OR "skill*" OR "technique" OR "decision making" OR "performance")) AND (sport*) | 153 |
| Web of Science  (1945 – Jan 2021) | Ts = (“mental fatigue” OR “mental exertion” OR “cognitive fatigue” OR “cognitive exertion” OR “mental exhaustion” OR “mental tiredness”) AND Ts = (“athletic performance” OR “technical skill*” OR “skill*” OR “technique” OR “decision making” OR “performance”) AND Ts = (sport*) | 83 |
| EBSCOhost  (1984 - Jan 2021)  (CENTRAL, SPORTDicus) | ( “mental fatigue” OR “mental exertion” OR “cognitive fatigue” OR “cognitive exertion” OR “mental exhaustion” OR “mental tiredness” ) AND ( “athletic performance” OR “technical skill*” OR “skill*” OR “technique” OR “decision making” OR “performance” ) AND sport* | 914 |
| Scopus  (2004 – Jan 2021) | (TITLE-ABS-KEY("mental fatigue" OR "mental exertion" OR "cognitive fatigue" OR "cognitive exertion" OR "mental exhaustion" OR "mental tiredness")) AND (TITLE-ABS-KEY("athletic performance" OR "technical skill*" OR "skill*" OR "technique" OR "decision making" OR "performance")) AND (TITLE-ABS-KEY(sport*)) | 92 |
