# Supplementary material for: Does mental fatigue affect skilled performance in athletes? A systematic review
Source: PLoS One. 2021 Oct 14;16(10):e0258307. doi: 10.1371/journal.pone.0258307 (PMC8516214; doi:10.1371/journal.pone.0258307)
Supplement: Attachment — Submitted filename: Response to Reviewers.docx [file pone.0258307.s003.docx]

Dear editor and reviewers,

Thanks for giving valuable feedback regarding the manuscript. We greatly appreciate it. We have been able to incorporate changes to reflect most of the suggestions. After thorough considerations, we respond to each point as below **two tables** (from the next page):

| **Table 1**  *Response to the Journal* | | | |
| --- | --- | --- | --- |
|  | **Jouranl Rquirement** | **Response** | **Page in the revised manuscript** |
| **1** | Meet the journal style requirements | Already checked formate | all pages |
| **2** | ORCID for corresponding author | linked |  |
| **3** | Include Table 4 mentioned in the manuscript | Sorry for the typo, actually it is Table 3 | 37-38 |
|  |  |  |  |
| **Table 2**  *Response to two reviewers* | | | |
| **Reviewer#1** | **Comments** | **Response** | **Page** |
| **General comments** | A thorough revision of the grammar and language of this manuscript is imperative before making further decisions on the acceptability of the manuscript. So please have the manuscript corrected by a native English speaker/a professional company for revising scientific documents. | The manuscript has been made proofreading by a professional service, for the authors are not native speakers. | all pages |
| **Specific comments** |  |  |  |
| **TITLE** | I suggest change to “Does mental fatigue affect skilled performance in athletes? A systematic review” | According to the definition of athlete, a person who is very good at sports, especially one who competes in organized events is an athlete. Therefore, the authors consider the comment and change the title to “Does mental fatigue affect skilled performance in athletes? A systematic review.” | 1 |
| **ABSTRACT** |  |  |  |
| **BACKGROUND** | Last sentence. Change “mechanism” for “effect”. | Yes, the reviewer is correct. The main contribution of the paper is the effect of mental fatigue on skilled performance, which has not been focused on by previous reviews. Although there is a discussion about the potential mechanism of impaired performance, the purpose is mainly about these effects. Thus, change “mechanism” to “effect.” | 1 |
| **CONCLUSION** | “Conclusion” could be dark and lined up to the left. | The format has been corrected | 2 |
| **INTRODUCTION** |  |  |  |
| **First paragraph** | Change “ball sports” to “team sports”. | *Thanks for the consideration of the reviewer, basketball and rugby as the example can be either ball or team sport. However, the sentence gives an example about skilled performance in sports, especially how to handle the ball. Also, the authors want to hold whole-length tightness with the “discussion” part, which emphasizes the cognitive demands in ball sports are more challenging, for athletes should predict trajectory paths of the ball (see details in the second paragraph of discussion). Therefore, authors have kept the term “ball sport”.* | 3  Please refer to discussion part: P17 |
|  | Change “Fernandez- Navarro et al7”to “Fernandez-Navarro et al. [7]” | changed | 3 |
|  | At the end of the first paragraph recommends for describe that fatigue can impair technical and decision-making skill. This information is importante because could link the first with second paragraph. | Accepted. The first paragraph is the background of the study about skilled performance. The second paragraph is the background of fatigue. The study was going to investigate the interaction of two aspects. Therefore, at the end of the first paragraph, it is better to describe fatigue influence performance in technical and decision making skill, which also can connect the second paragraph. | 3 |
| **Third paragraph** | Very long sentence “Since these executive functions are highly correlated to a series of actions, for example the capacity of initiating and stopping, monitoring and changing behaviour, and planning for subsequent moves [15], which is the key to a good sports performance among athletes [16-18], researchers recently started to investigate the effect of mental fatigue on soccer performance.” It’s recommended to divide it without the information being altered. | The long sentence was revised. | 4 |
| **Forth paragraph** | Change “ball game” to “team sport” | To hold whole-length tightness, authors have kept ball sport (reason see the italics). | 4 |
|  | Before sentence “To the authors’ knowledge, a comprehensive review on skilled performance in the context of mental fatigue is still unavailable.” the authors could describe the possible motives of mental fatigue impair the inhibitory control and consequently perceptual-cognitive skills (e.g., increased adenosina in ACC). | Agreed and added one sentence to describe mental fatigue may influence inhibition of executive functions. | 4 |
| **Seventh paragraph** | Change “on performance in endurance performance” to “on endurance performance” | Revised the paragraph | 6 |
|  | Change “ball sports” to “team sports” | Revised the paragraph | 6 |
|  | Although the most adopted framework model in the literature of mental fatigue is psychobiological, this model does not seem to explain the changes in perceptual-cognitive skills (e.g., decision-making). Perhaps the authors could explain the brain mechanisms pointed by Terry McMorris (e.g., attenuated dopamine to front-parietal regions) and visual search strategy (visual cortex) changed in athletes mentally fatigued | Agreed and revised paragraph | 6 |
| **METHODS** | It’s recommended add “data analysis” for describe the subgroups created in results section: Technical skill, offensive skill, defensive skill, and decision-making skill | added the “data analysis.” | 8 |
| **RESULTS** |  |  |  |
| **“Overview of Sports”**  **First paragraph** | Change “ball sports” to “team sports” | Keep “ball sport” (reason see the above italics). | 12 |
|  | intervention inducing and manifestation of mental fatigue It’s suggested to create a Table indicating the main findings (e.g., task used to induce mental fatigue, test or scale utilized for measurement mental fatigue) | Originally, there are tasks to induce mental fatigue (see the column of Manipulation in Table 3). Follow recommends and to explain clearer, added the measurement of mental fatigue in all extracted studies (Table 3). | See Table 3: 37-38 |
| **DISCUSSION** |  |  |  |
| **Intervention inducing and manifestation of mental fatigue** |  |  |  |
| **Second paragraph** | “However, contrast to the study of [50], the higher perception of effort is not only responsible for the impairment in physical performance, but also skilled performance. Four selected literature have measured the effects of motivation as part of the methodology [36, 40, 42, 44].” The sentences are unconnected. It’s recommended re-writing. | Added more information obtained from results about the manifestation of mental fatigue. And the original second paragraph was deleted, for it is unconnected. | 20-21 |
|  | I understanding that the impaired perceptual-cognitive skill in athletes mentally fatigue can be explain by visual search strategy altered or by activity changed in front-parietals regions (e.g., increased theta wave in PFC). It’s suggested describe about this. | Accepted. The potential mechanism is discussed in the new subtitle “**Potential mechanism**.” Identified specific executive functions of technical and decision making skill and discussed literature from cognitive science on the effects of mental fatigue on these executive functions**.** | 24-26 |
| **IMPLICATION** |  |  |  |
| **First paragraph** | It’s recommend to remove the first paragraph, because the findings of systematic review no demonstrated rating perceived exertion as a mediator between mental fatigue and skilled performance. | Accepted. The original paragraph was removed and took the place of potential mechanisms of impairment. | 27 |
| **Second paragraph** | There is a difference between rating perceived exertion (RPE) and session-RPE. For evaluate session-RPE is necessary measure RPE 30-min after physical or experimental session. Some studies measured RPE after 30-min for analyze session-RPE or internal training load. It’s suggested review this paragraph. | Accepted. Removed some sentences about RPE, emphasizing the impact of duration after cognitive tasks, which is more related to that paragraph. | 27 |
| **Reviewer #2** |  |  |  |
| **General Comments** | This is a timely systematic review of the effects of mental fatigue on skilled performance in sport which I believe can have a good impact in the field. There are, however, several issues to be resolved before publication as explained in the specific comments. I also suggest the authors to have the whole manuscript reviewed by an English copyeditor as some terminology and phrases are often unusual and odd. Some parts are also unnecessary and can be eliminated (see specific comments) | The manuscript has been made proofreading by a professional service, for the authors are not native speakers. | all pages |
| **Specific comments** |  |  |  |
| **INTRODUCTION** | Although not entirely similar, the authors should include in their introduction and discussion reference to the literature review by Habay and colleagues 1 dealing with the effects of mental fatigue on psychomotor performance, including tests of skilled sport performance | Added at the 6^th^ paragraph in the **“Introduction”** to describe the study of Habay et al. (2021). | 5 |
|  | The paragraph on the single narrative review on sport-related performance can be eliminated or shortened | Accepted. The paragraph on the single narrative review on sport-related performance was eliminated. | 5-6 |
|  | The psychobiological model proposed by Marcora is a model of endurance performance and cannot be transferred so easily to skilled sport performance. Although you can mention the (generally lack of) effect of mental fatigue on motivation, the most relevant explanations for the negative effects of mental fatigue on skilled sport performance are its effects on attention, inhibitory control, and other executive functions. In the context of skilled sport tasks, RPE is more an index of how much effort the athletes exerted during the tasks (which can be similar but with different performance outcomes due to mental fatigue) rather than a mechanism of impaired performance. So I suggest reducing the sections about the psychobiological model of endurance performance. | Agreed and the last paragraph in **“Introduction”** was re-writing, reducing the section of psychobiological. | 6-7 |
| **METHODS** | One of the main methodological problems of this paper is the lack of proper operational definitions of the PICO criteria. Please provide in the revised manuscript. | Added operational definitions of PICO, in Methodology: Eligibility Criteria, and some categories. | 7-8 |
|  | There should also be operational definitions for the categories you have used to analyse the results of the systematic review, e.g. level of the athletes, difference between offensive and defensive skills, etc. | Accepted. Skilled performance as the primary outcome in the current review was highlighted and put separately at Methodology: Data analysis. | 8 |
|  | Fnally, you should also include information about the dates and language limits you used for the search. | The original manuscript shows the limits of language (English: see **Eligibility criteria** first paragraph: Page 7**)** and dates (the inception dates to January 2021: see **Methodology** first paragraph: Page 7). | 7 |
| **RESULTS** | In general, in the text I would like to see more quantitative information about the literature, for example absolute values and/or percentages of papers in relation to the analyses shown in Table 3. | Agreed and added absolute value and percentage in results | 16-18 |
| **DISCUSSION** | Try not to repeat too much the results in the Discussion. Instead summarise and then compare the observed effects of mental fatigue with the effects of other related factors, e.g. i) the decline in technical performance between the first and second half of a soccer match, ii) the effects of sleep deprivation or iii) the effects of physical fatigue. | It is a good idea to compare the observed effects of mental fatigue with the effect of other related factors, therefore, the authors discussed the duration of the mental fatigue-inducing task and the types of subsequent performance tests toward skilled performance. However, the Authors did not discuss other factors, for the information in the extracted literature is not comprehensive.  **Discussion was revised (see the below content).** |  |
|  | I also suggest that, for each skilled sport performance task, you identify some key executive functions and discuss literature from cognitive science on the effects of mental fatigue on these executive functions. Such discussion would provide a much better explanation of potential mechanisms than the current explanation based on the psycholobiological model which needs to be reduced in length and de-emphasised as the main explanation for the negative effects of mental fatigue on skilled sport performance (see also comments about the Introduction). | Accepted and revised discussion part mainly from below 2 points:  (a) reduced write-up part about the psychobiological model, instead of identifying key executive functions from technical and decision making skill. Also, discussed cognitive science literature on these executive functions (see details in “**Potential mechanism**”**)**  (b) discussed more **“Intervention inducing and manifestation of mental fatigue”** based on corresponding results. | (a) 24-26  (b): 19-21 |
